# Fabrication of in-situ rod-like TiC particles dispersed Ti matrix composite using graphite power sheet

**DOI:** 10.1038/s41598-022-23796-4

**Published:** 2022-11-09

**Authors:** Ning Wang, Yongbum Choi, Kentaro Oue, Kazuhiro Matsugi

**Affiliations:** 1 Department of Mechanical Science and Engineering, Graduate School of Engineering, Hiroshima University, 1-4-1 Kagamiyama, Higashi-Hiroshimasi, Hiroshimaken 739-8527 Japan; 2 Mechanical Engineering Program, Graduate School of Advanced Science and Engineering, Hiroshima University, 1-4-1 Kagamiyama, Higashi-Hiroshima, Hiroshimaken 739-8527 Japan

**Keywords:** Engineering, Materials science

## Abstract

Titanium matrix composites (TMCs) with TiC reinforcements were fabricated by an in-situ method that evolves pure titanium foils (thick: 100 μm) and graphite powder sheets by spark plasma sintering. 20 μm thick graphite powder sheets with PVA (polyvinyl alcohol) were fabricated as carbon resources. The effects of different sintering temperatures and heating time on microstructural features, interface, and properties of the composites were investigated. The structural and microstructural analyses were performed by EPMA, FE-SEM, and EDS. The XRD patterns taken from the cross-section of the prepared composites revealed the composites are composed of TiC_x_ and hexagonal close-packed (HCP) α-Ti. Homogeneous rod-like TiC_x_ particles reinforced TMCs were evaluated by tensile property. The tensile properties of the rod-like TiC_x_-reinforced TMC show that the tensile strength (UTS) is 479 Mpa, which is 81.4% higher than pure titanium. The formation mechanism and enhancement mechanism of rod-like TiC_x_ particles are also discussed.

## Introduction

Titanium matrix composites (TMCs) as lightweight structural materials have received a lot of interest in the last decades by reason of their excellent properties, such as high elastic modulus, high strength, good creep, and fatigue resistance even at elevated temperatures^1–6^. Therefore, it has a broad application prospect in many fields such as the aerospace industry, biomedical and healthcare industry, the energy, and power generation industry and the petrochemical industry^1–4^. However, Ti base materials will gain substantial prospects for more widespread application if their properties can be improved beyond current Ti alloys and processing technologies. It is possible to produce TMCs with more competitive cost and performance, which will have great prospects in the field of replacing other metallic materials^2–4^. Therefore, researchers have conducted a lot of exploration on titanium matrix composites to provide better properties and performance.

As well known, it has been found that the mechanical properties of TMCs mainly depend on the composition and microstructure of the matrix and reinforced particles^7^. The in-situ synthesis method refers to the synthesis of reinforcing materials in the matrix by utilizing the reaction between the reactants and the alloy matrix. The in-situ fabrication technique is considered to be one of the most promising methods for preparing titanium matrix composites in the matrix due to the fine size and the strong interfacial bonding between the matrix and the reinforcement phase^3^. Many high-modulus ceramic reinforcements for TMC have been investigated, such as SiC, TiB, Al_2_O_3_, and TiC. Among them, TiC is considered as the most promising reinforcement material due to its excellent chemical compatibility with Ti matrix^5,9^. TiC is a transition metal carbide with a face-centered cubic (FCC) NaCl-type structure. Its true composition is often non-chemometric and is represented by the general formula TiC_x_. Here x is the ratio of C to Ti, which ranges from 0.46 to 0.98^8–10^. Conventional methods of preparing TMCs are mostly mechanical alloying (MA), powder metallurgy (PM), and ingot metallurgy technique (IM) with both advantages and disadvantages, uneven distribution of TiC particles, extremely high sintering temperature and complex heat treatment procedures are considered major drawbacks^11,12^. Generally, laminated sintering is used in the preparation of composites with micro-laminated structures, which has the advantages of uniform distribution, simple preparation processes and strong interfacial bonding^13^. In this study, TMCs were fabricated by hot press sintering on alternating stacks of titanium foils and graphite powder sheets. In order to completely diffuse the graphite layer into the titanium matrix, ultra-thin graphite powder sheets made of graphite powder and PVA were used as a carbon source for the laminated sintering. In addition, the microstructural evolution during the fabrication of in-situ TiC-reinforced TMCs was investigated with a particular focus on the formation mechanism of rod-like TiC particles. Tensile tests were carried out as an assessment of the mechanical properties.

## Materials and experimental

### Starting materials

Pure Ti plates (> 99.49%, 1 mm thick), pure Ti foils (> 99.49%, 0.1 mm thick), graphite powder (> 90%, 5 μm), and PVA solution (polyvinyl alcohol, 13 wt%) were used as starting materials. The detailed properties of pure titanium are listed in Table 1.

**Table 1:** Properties of pure titanium.

Thickness *t* (mm)	Purity (%)	Tensile strength *σ*_*TS*_/ (MPa)	Yield strength *σ*_*YS*_/ (MPa)	Elongation δ (%)
1	> 99.49	320	165	27

### Fabrication of graphite powder sheet

**Figure 1:**
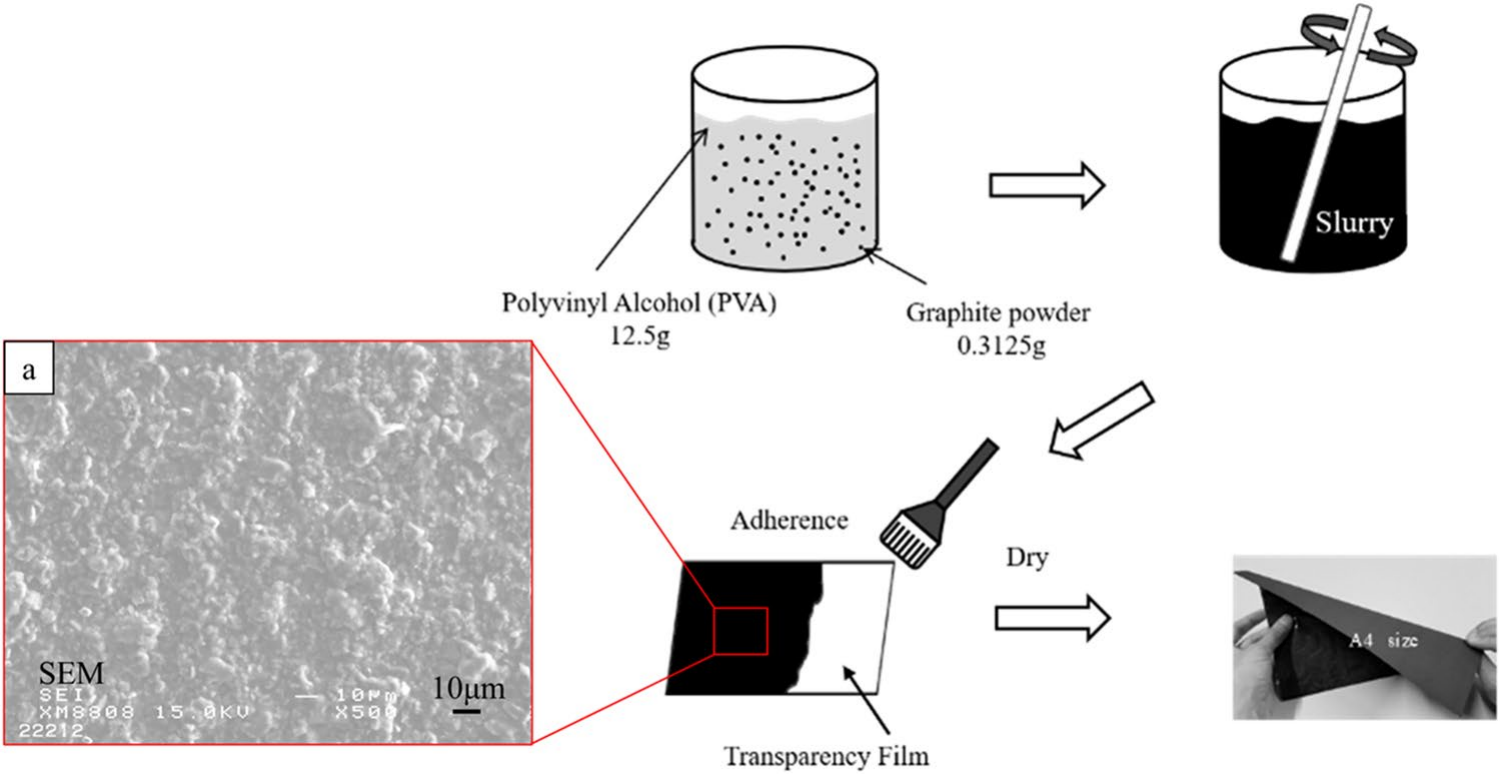
Schematic illustration of fabricating processes of Graphite powder sheet: (**a**) the SEM image of graphite powder sheet.

The graphite powder sheet as the carbon source was prepared as follows (shown in Fig. 1): 0.3125 g graphite powders were mixed in 12.5 g PVA solution (13 wt %) in a beaker and continuous stir at 323 K for 1 h to form a homogeneous solution. The resultant solution was rolled evenly on a transparency film and then put in a drying oven to evaporate the solvent at 343 K for 24 h. The thickness of the resultant graphite powder sheet is about 20 μm. As shown in Fig. 1a, the graphite powder sheet shows smooth and uniform in texture.

### Reaction of single layer of graphite powder sheet and Ti plates

Two Ti plates and one graphite powder sheet were cut into ɸ10 mm disks and then use fine quartz sandpaper to remove the oxide film on the surface of Ti plates. One piece of graphite powder sheet was sandwiched by two pieces of Ti plates and then sintered in a spark plasma sintering (SPS) furnace at 873 K, 973 K, 1073 K, 1173 K, and 1273 K, respectively, under 50 MPa for 0.6 k s to investigate the amount of reaction production (TiC) between graphite powder sheets and Ti plates with different sintering temperature.

### Fabrication of TMCs with multi-layer graphite powder sheets

**Figure 2:**
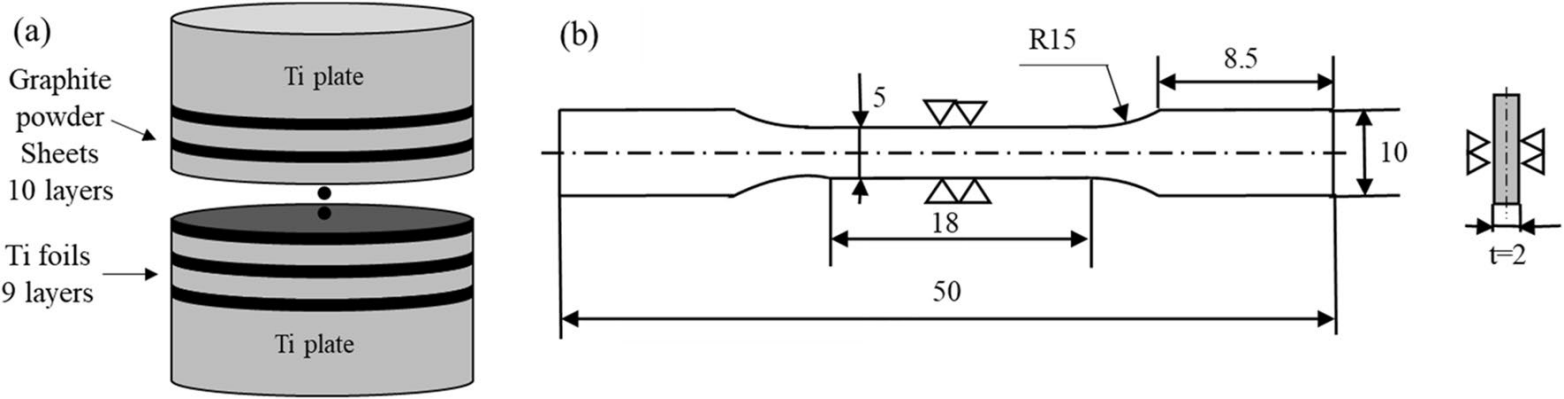
(**a**) Schematic illustrations of preparation processes of large-scale samples; (**b**) Image of shape of tensile specimen (ASTM test method E8M-11, unit: mm).

TiC reinforced Ti matrix composites were prepared in a similar way as above. Ti plate, Ti foils, and graphite powder sheets were cut into ɸ10 mm disks and then the Ti plates and foils were cleaned in the ultrasonic bath of acetone for the 1.2 k s. Then, two thick Ti plates are placed on the top and bottom ends, alternating between one piece of graphite powder sheet and one pieces of titanium foil in the middle as shown in Fig. 2a. A total of 10 pieces of graphite powder sheet and 9 pieces of titanium foil were used. The mass fraction of graphite is 0.82 wt %. LBN spray (ingredients: methyl ethyl ketone, dimethyl ether, isopropyl alcohol, nitrocellulose, manufactured by Showa Denko) was used as release agent. And then the samples were sintered in a spark plasma sintering (SPS) at 1273 K under 50 MPa for 0.6 k s. To investigate the effect of sintering temperature and heating time, the experiment of sintering at 1473 K for 0.6 k s and 1473 K for 3.6 k s was conducted. Microstructures of composites are revealed by Scanning Electron Microscope (SEM, TOPCON SM-520, Japan). The powder morphology and sintered microstructure were observed by Electron Probe Micro-Analyzer (EPMA, JXA-8900, Japan). X-ray diffraction (XRD; D/max-2500/PC, Japan) analysis was carried out using Cu Kα radiation (*λ* = 1.54056 Å) at a scanning speed of 1° /min over the 2*θ* range of 30°­–75°. A hydraulic servo strength tester (SHIMAZU, EHF-LV020K1-020) is used for the tensile test of composite materials. Tensile test conditions are based on ASTM test method E8M-11, crosshead speed 0.5 mm / min. Strain gauge F-02 W-12T11W3 (strain limit 2%, manufactured by Minebea Co., Ltd.) for Young's modulus measurement. The shape of the test piece of the tensile tester is shown in Fig. 2b. The fracture surface of the test piece after the tensile test is observed using SEM.

## Results and discussion

**Figure 3:**
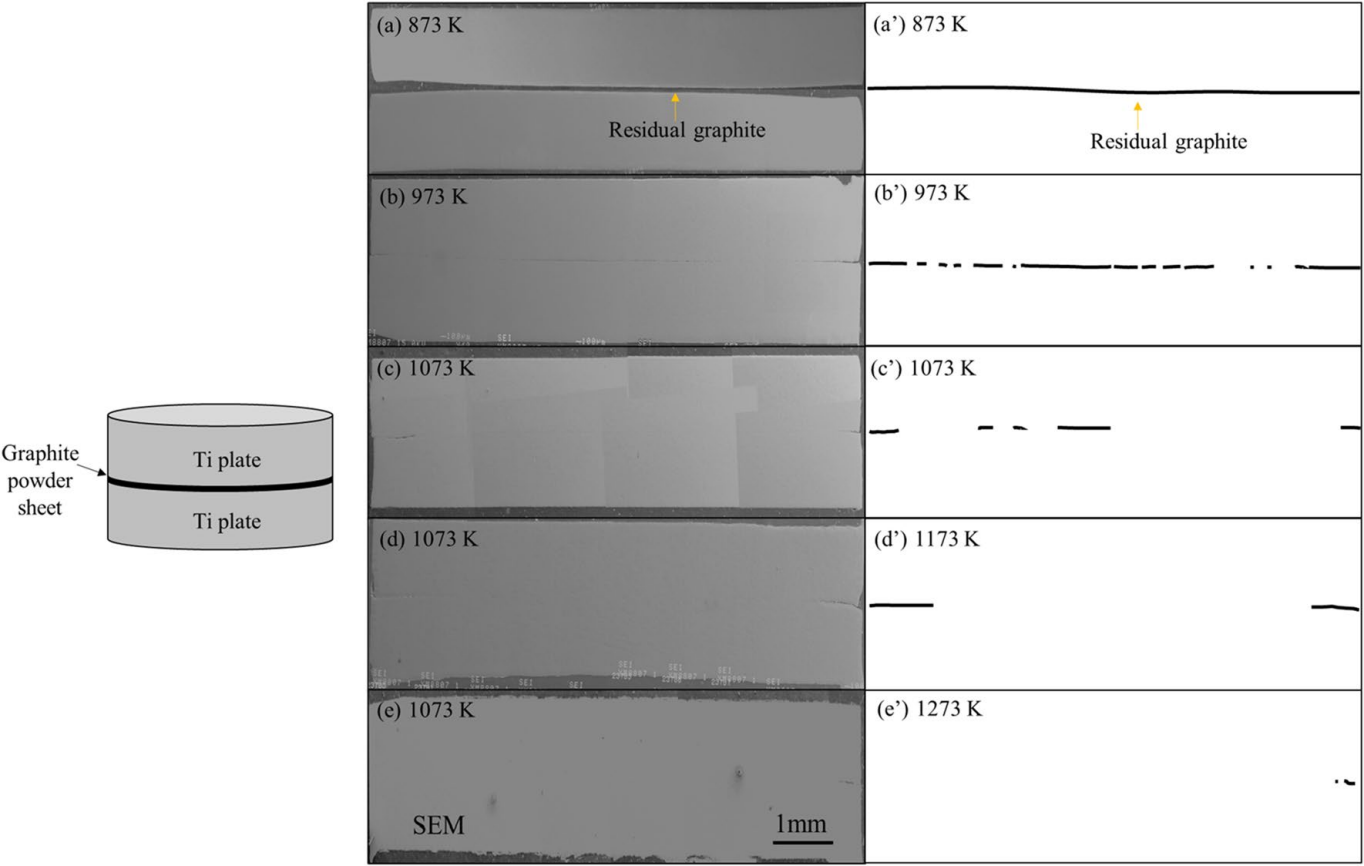
SEM images of Ti matrix composite by different of sintering temperature: 873 K(**a**), 973 K(**b**), 1073 K(**c**), 1173 K(**d**) and 1273 K (**e**); (**a**’)-(**e**’): Corresponding replication view of the residual graphite.

**Figure 4:**
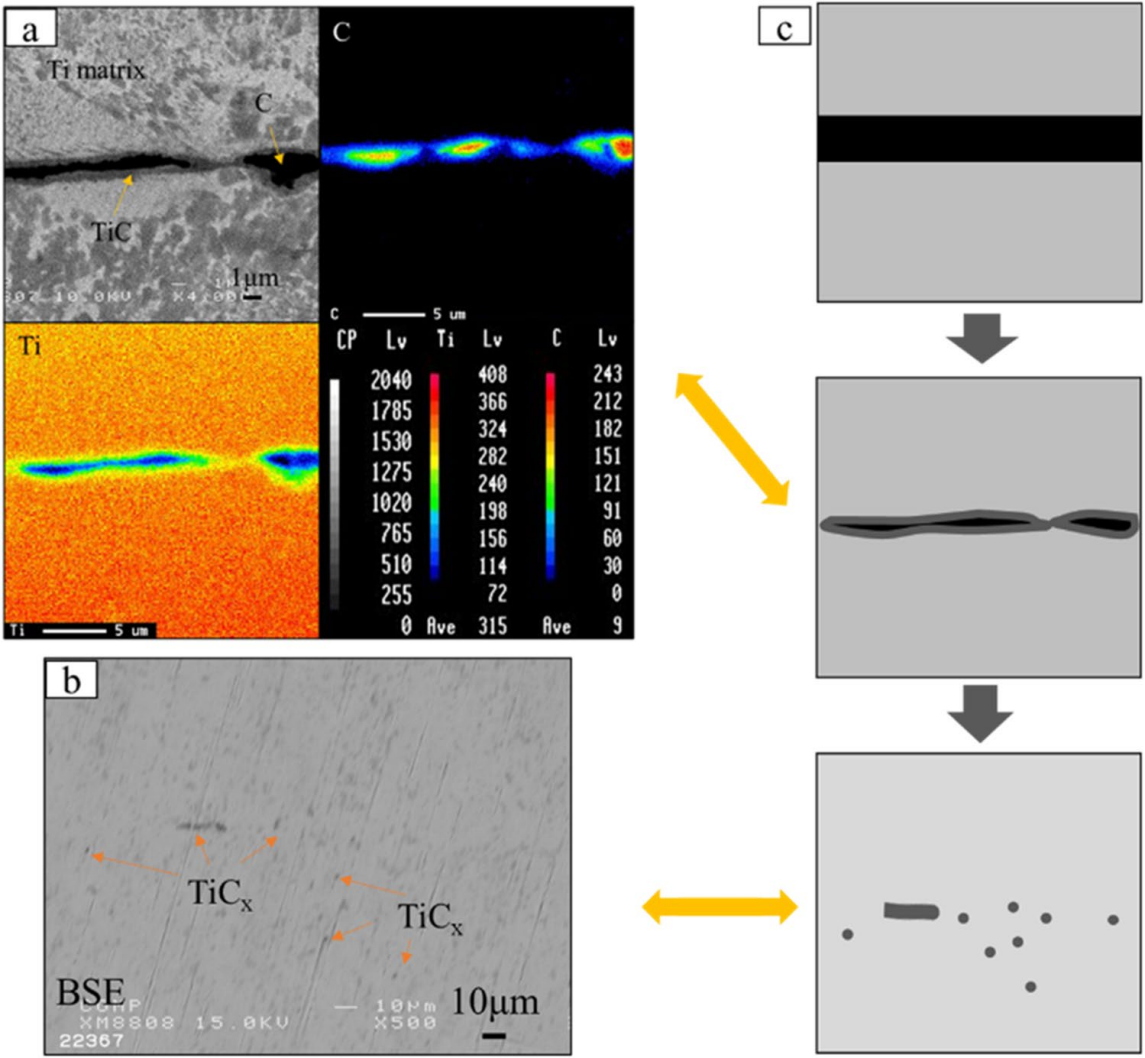
(**a**) BSE image and area mapping by EPMA of bonding area of TMC of 1073 K; (**b**) BSE image of TMC of 1273 K; (**c**) Schematic illustration of synthetic process of in-situ TiC particles dispersed TMC.

**Figure 5:**
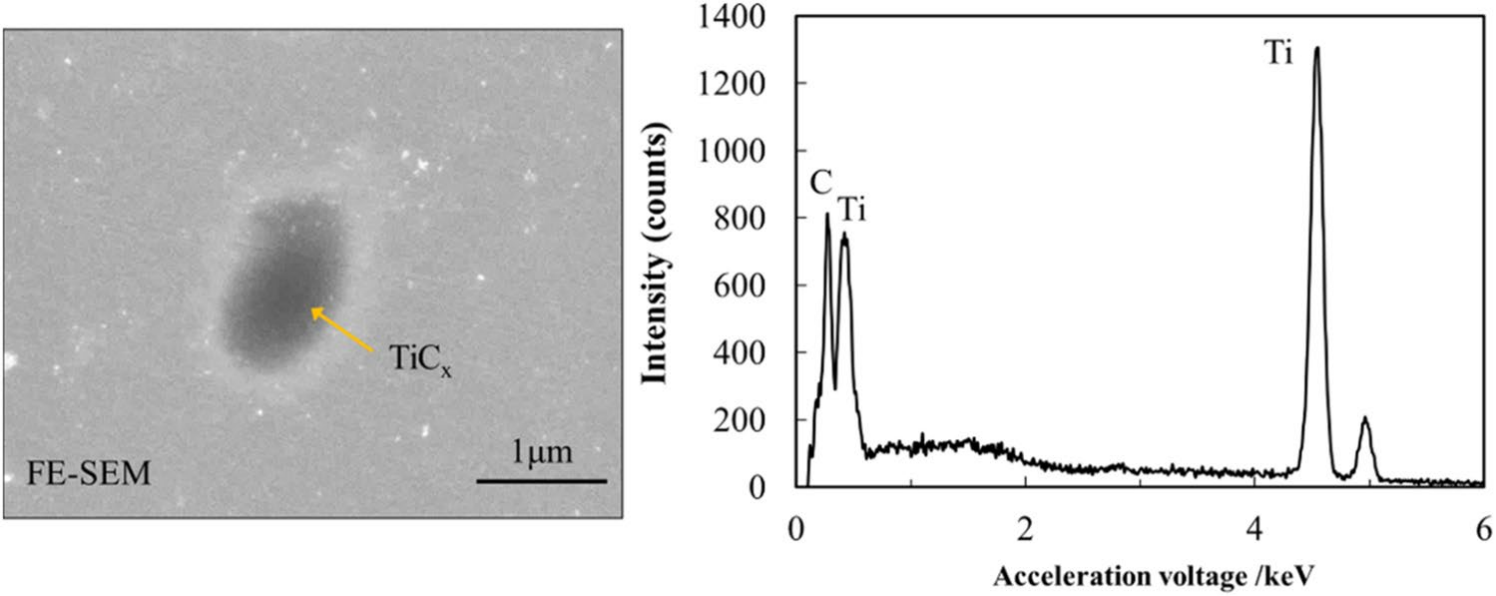
FE-SEM image and point analysis by EDS of TiC reinforced TMC sintered at 1273 K.

To observe the process of titanium-carbon diffusion reaction, a set of samples with different sintering temperatures was prepared. Figure 3a–e shows the vertical section of single-layer Ti matrix composite. Figure 3a’–e’ are replication views of the residual graphite sheet after sintering. At the sintering temperature of 873 K, almost no reaction was observed at the bonding area between the two titanium plates and the graphite powder sheet. At the sintering temperature of 973 K and above, it can be observed macroscopically that the titanium plates bonded together, and the bonding area increased as the sintering temperature increased. When the sintering temperature was increased to 1273 K, about 95% of the graphite sheet reacted with titanium plates. At microscopic view, the reaction process is shown in Fig. 4. BSE image and area mapping of the sample with sintering temperature of 1073 K is shown in Fig. 4a. A dark gray interlayer can be seen at the interface of remained graphite and titanium plates, which is considered to be TiC. In some places, two titanium plates bond together, which is probably caused by the uneven thickness of graphite powder sheet. When the sintering temperature was increased to 1273 K, as shown in Fig. 4b, most of the graphite layer disappeared, but some short gray lines were observed at the original graphite powder sheet positions. In addition, many gray particles interspersed around the original graphite powder sheet position, but there are few particles presenting away from the center. To analyze the gray particles formed in the matrix, an FE-SEM image and EDS point analysis on these gray particles are shown in Fig. 5. As the results of point analysis, the dark gray particles are determined to be TiC_x_ particles. These TiC particles have an average particle size of 1 µm. Besides, the standard free energy ΔG of TiC formation was calculated by following equation when the sintering temperature is lower than 1973 K:

1 $${\Delta G} = - 184571.8{ } + 41.382T - 5.042T{ }lnT + 2.425{ } \times 10^{3} { }T^{2} - 9.79 \times { }10^{5} /T{ }(T{ } < 1939K){ }$$

The Gibbs free energy ΔG for the reaction of titanium and graphite at 1073 K and 1273 K can be calculated as -172 kJ/mol and -174 kJ/mol, respectively^14,15^. This result indicated the formation of TiC_x_ at 1073 K and 1273 K is spontaneous generation. Figure 4c is a schematic diagram of the diffusion–reaction between graphite and titanium matrix with increasing temperature.

**Figure 6:**
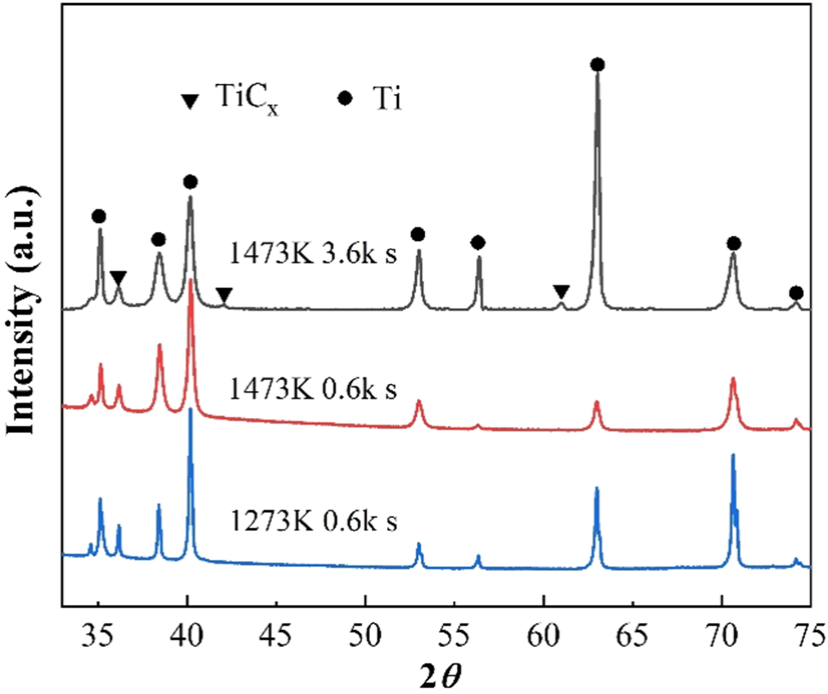
XRD pattern of TiC reinforced TMC sintered with different sintering temperature and heating time.

**Figure 7:**
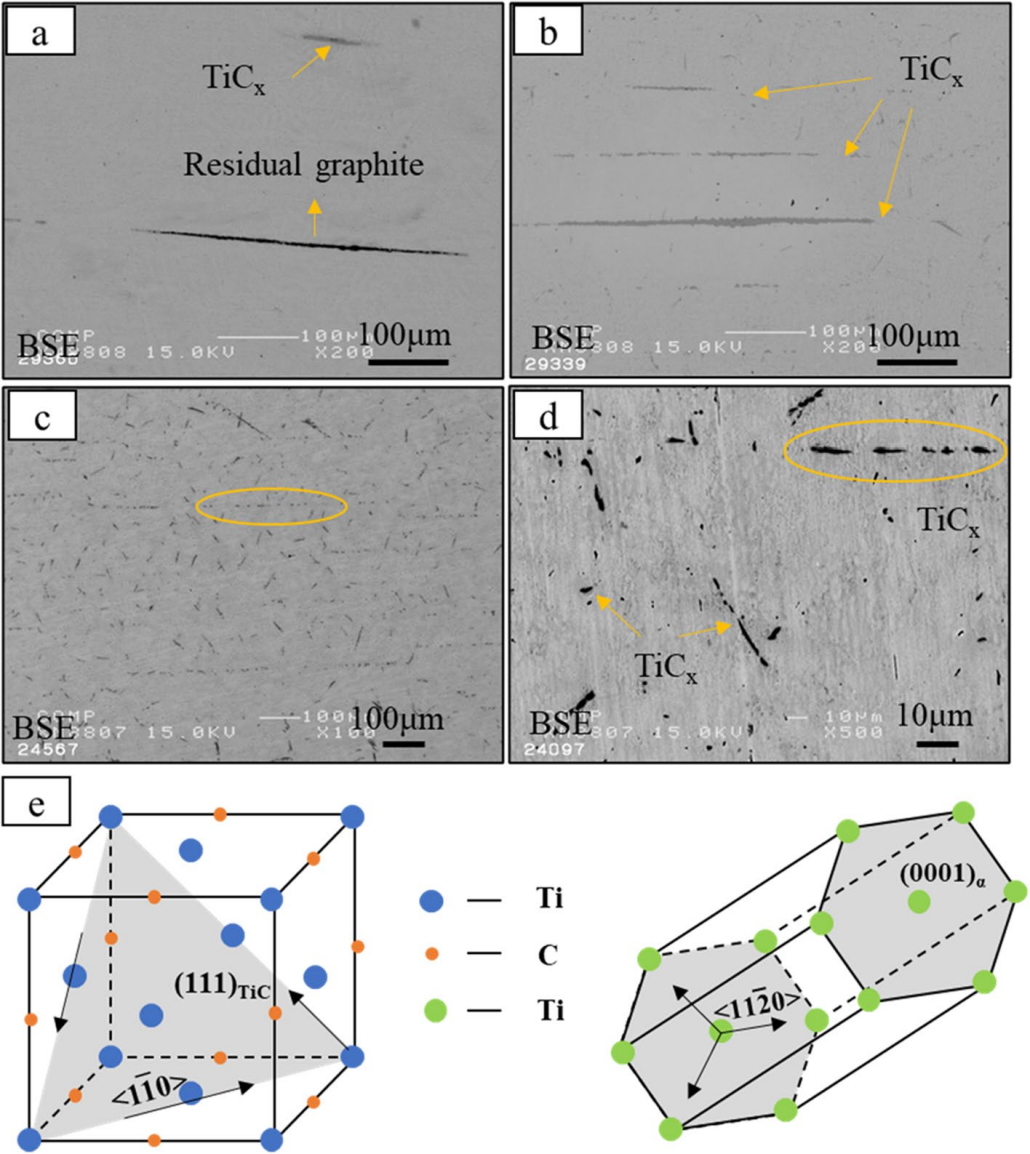
(**a**) BSE images of TiC reinforced TMCs sintering at 1273 K for 0.6 ks; (**b**) 1473 K for 0.6 ks; (**c**) and (**d**) 1473 K for 3.6 ks; (**e**) The schematic diagram of the ORs of TiC and α-Ti.

In order to investigate the mechanical properties of composites, large-scale multi-layered specimens of in-situ TiC reinforced TMCs were fabricated. Several layers of graphite powder sheets and titanium sheets were used as starting materials (as shown in Fig. 2). Figure 6 shows XRD patterns of large-scale samples with different sintering temperature and heating time. It is evident from the Figure that each composite consists of TiC_x_ and hexagonal close-packed (HCP) α-Ti. The sample with sintering temperature of 1473 K for 3.6 k s shows the biggest diffraction peak intensity. Figure 7 shows the microstructure of the large-size TiC reinforced TMCs. Figure 7a is the sintering temperature of 1273 K for heating 0.6 k s. For this sample, there are both dark lines and gray lines were observed. The dark line is considered to be residual graphite and the gray line is considered to be the TiC_x_ layer formed at the bonding area of Ti foils and graphite powder sheet. In Fig. 7b, by keeping the holding time constant for 0.6 k s and increasing the sintering temperature to 1473 K, there are no deep dark lines were observed instead a gray line appeared in the original position. It demonstrates the graphite has completely reacted with Ti matrix at 1473 K. Figures 7c,d are the samples of sintering temperature of 1473 K for 3.6 k s. In this sample, no obvious thick long lines were formed, instead numerous rod-like TiC_x_ particles were observed in the matrix and homogenous composite was obtained. In Fig. 7b, due to the short holding time, the C atoms in the graphite layer have not completely diffused into the Ti matrix, resulting in a locally high concentration of C atoms. Therefore, a layer-like TiC_x_ forms at the interface. When the holding time is increased to 3.6 ks, the sample is closer to thermodynamic equilibrium and most of the carbon atoms diffuse uniformly throughout the sample^16,17^. However, at the original position of graphite powder sheets, straight dotted lines formed by the arrangement of the short rod TiC can be still observed. The solid solubility of carbon atoms in titanium matrix is 0.05 wt% at room temperature, and 0.13 wt % above 1193 K. Figure 7e shows the schematic diagram of the ORs of TiC_x_ and α-Ti. The mechanism of formation of rod-like TiC_x_ particles were discussed in the following. Previous studies have already indicated the α-Ti co-deposited with TiC follows the crystallographic relationship: (0001) _α_ // (111) _TiC_; [11_20] _α-Ti_ // [110] _TiC_, in accordance with the TiC (111) preferred orientation^8,18^. As shown in Fig. 7c, the short rod-shaped TiC_x_ exhibits a regular angle to the direction of the Ti foils, at around 45 degrees. This is thought to be the carbon atoms precipitating out of the titanium matrix and growing in a selective orientation along the (0001) _α-Ti_// (111) _TiC_. When the temperature is lower than 1155 K, the atomic structure of titanium is HCP, and the solubility of carbon atoms in titanium is ~ 0.05 wt%^19^; when the temperature is higher than 1155 K, the atomic structure of titanium is body-centered cubic (BCC), and the solubility of carbon atoms in titanium is ~ 0.15 wt%. When the temperature rises to 1153 K, the C atoms are dissolved from the graphite powder sheets. Part of the C atoms diffuse into the Ti matrix. When the temperature falls below 1153 K, the C atoms precipitate due to the reduced solubility of the C atoms, and the precipitated C atoms diffuse into the titanium octahedral voids along the (0001) _α-Ti_// (111) _TiC_ in a selective orientation, forming TiC_x_. In addition, many TiC_x_ particles with an average diameter of about 1 µm can also be observed in Fig. 7c,d, which is similar to the particles in Fig. 4b. TiC_x_ particles with two morphologies are present in the TMC prepared made from laminated stacks. Previous studies have demonstrated that the final shape of in situ TiC_x_ is directly related to the value of x^20^. In C-atom vacancy structures, the number of C atoms is less than in structures without vacancies. Therefore, during the formation of TiC_x_ crystals, the low C contributes to the atomic vacancies. As a result, it does not grow into a cube, but into a sphere^20,21^. Therefore, when the value of x turns low, the TiC particles do not grow into short rods, but into sphere particles.

**Figure 8:**
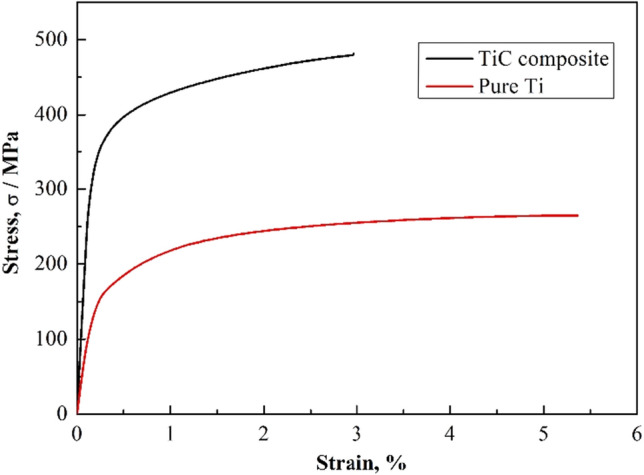
Tensile strength-displacement curve of of pure Ti and TiC reinforced TMC at 1473 K for 3.6 k s.

**Figure 9:**
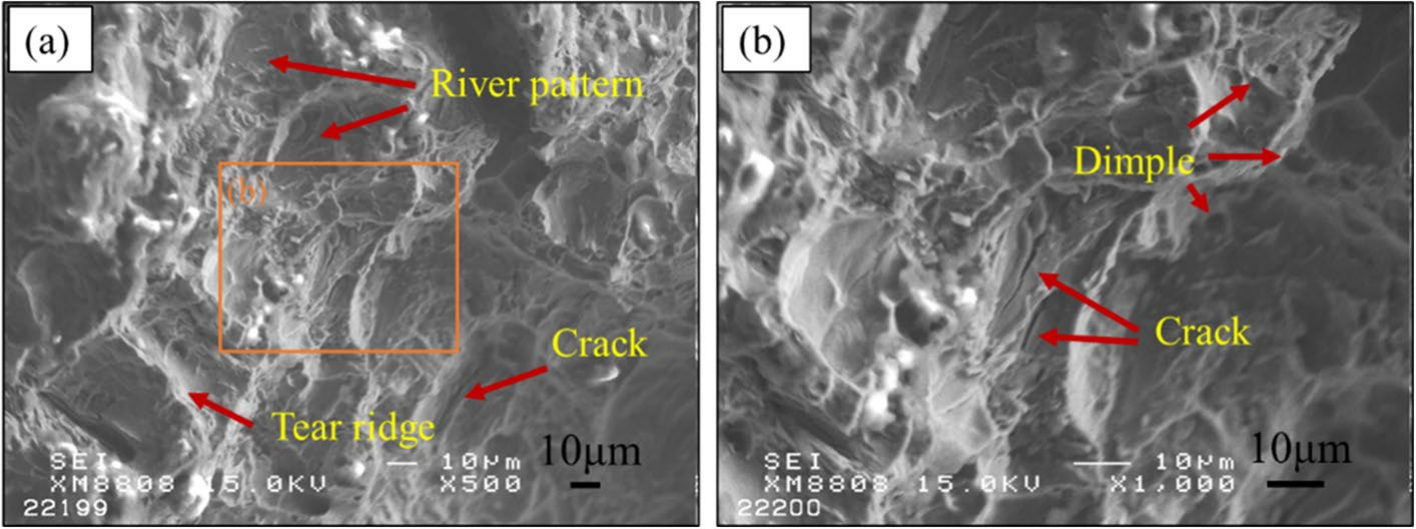
(**a**) SEM images of fracture surface of TiC reinforced TMC at 1473 K for 3.6 k s; (**b**) magnified image.

Figure 8 shows the tensile stress–strain response curves for pure titanium and 1473 K TMC over 3.6 k s. The rod-like TiC_x_ reinforcement improves the strength of the titanium matrix but reduces its ductility compared to pure titanium. The composites showed significant improvements with ultimate tensile strength (UTS) of 479 MPa, yield strength (YS) of 289 MPa and elastic modulus (E) of 234 GPa, 81.4%, 140.8% and 120.7% higher than that of pure Ti, respectively. These enhancements came at the cost of ductility, which was reduced by 49.0%. Figure 9 shows the fracture diagram of the rod TiC_x_-reinforced TMC sample after tensile testing. As shown in Fig. 9a, the composite shows typical ductile fracture characteristics with some river patterns and tear ridges clearly visible on the fracture surface. Some cracks can be observed in the corresponding magnified image of Fig. 9b. This observation suggests that the applied load may have been transferred from the substrate to the TiC_x_ particles, since strong interfacial adhesion can provide effective load transfer capability and thus increase the yield strength^17^. Moreover, there are many ununiform dimples could be observed, which indicating good plasticity. Strengthening mechanism of rod-like TiC_x_ particles in Ti matrx were discussed in the following. TiC_x_ particles have excellent mechanical strength with an elastic modulus of 450 GPa, which is much higher than that of pure Ti (106.4 GPa). When load is applied to the composite, the hard TiC particles are able to bear part of the load. As shown in Fig. 9a, It shows a brittle fracture manner in the TiC_x_ particles and ductile manner in the matrix. In addition, the in-situ synthesized TiC_x_ particles have strong interfacial adhesion with the Ti matrix, which ensures an effective load transfer capability, thereby increasing the yield strength^18^. As shown in Fig. 9b, strong bonded interfaces between Ti and TiC transfer loads without debonding. In our work, the addition of a small amount of carbon (0.82 wt %) resulted in a significant increase in the tensile strength of the titanium matrix. However, the tensile properties of our rod-like TiC_x_-reinforced TMCs are not outstanding when compared to the reference literature. For instance, Castro et al.^22^ reported a 0.21 vol.% TiC-reinforced TMC by ingot casting metallurgy technique, with UTS of 565 MPa, which correspond to an increase of 47.4%, compared to pure Ti (UTS of 386 MPa). Lu et al.^23^ reported a 3 wt.% PCS (polycarbosilane) of TiC-reinforced TMC by powder metallurgy, with UTS of 861 MPa, which correspond to an increase of 56.8%, compared to HDH (hydride-dehydride) pure Ti (UTS of 549 MPa). In contrast, although the UTS of our rod-like TiC reinforced TMC was 479 MPa, it significantly increased by 81.4% compared to the pure Ti (UTS of 264 MPa). As well known, the tensile properties of composites are also highly dependent on the matrix. Therefore, in future research, we will focus on improving the tensile properties of rod-like TiC_x_ reinforced TMCs for example by changing matrix materials.

## Conclusion

(1) Graphite powder sheet and titanium plates were bonded together due to the solid phase reaction over 973 K. The bonding area increased as the temperature increased. The bonding rate was up to 95% at sintering temperature of 1273 K. TiC_x_ particles with an average diameter of 1 µm were observed in the Ti matrix nearby the original position of graphite powder sheet.

(2) For large samples, a homogenous rod-like TiC_x_ particles reinforced TMC using pure titanium foils and graphite powder sheet was fabricated by increasing the sintering temperature and holding time to 1473 K for 3.6 k s. XRD indicated all composites are composed of TiC_x_ and hexagonal close-packed (HCP) α-Ti.

(3) The rod-like TiC_x_ particles reinforced TMC have improved mechanical properties compared with pure titanium. The result of tensile test revealed the tensile strength of 479 MPa, yield strength of 289 MPa and elastic modulus of 234 GPa, which were 81.4%, 140.8% and 120.7% increased, respectively.

## Data Availability

The datasets generated and analyzed during the current study are available from the corresponding author on reasonable request.
